# The impact of technical factors on the chest radiographic findings in COVID-19 pneumonia

**DOI:** 10.4102/jcmsa.v4i1.313

**Published:** 2026-07-21

**Authors:** Zukiswa Sobetwa, Stephanie Griffith-Richards, Martin Kidd, Richard D. Pitcher

**Affiliations:** 1 Division of Radiodiagnosis, Department of Medical Imaging and Clinical Oncology, Faculty of Medicine and Health Sciences, Stellenbosch University, Cape Town, South Africa; 2 Centre for Statistical Consultation, Stellenbosch Business School, Stellenbosch University, Cape Town, South Africa

**Keywords:** COVID-19, chest X-ray, SARS-CoV-2, chest radiography, CXR severity scoring systems, technical factors on chest x-ray, South Africa

## Abstract

**Background:**

Published coronavirus disease 2019 (COVID-19) chest X-ray (CXR) severity scoring systems do not consider the impact of technical factors on radiographic findings. This study aimed to describe the technical features of COVID-19 pneumonia CXRs, determine their impact on CXR interpretation and reappraise published COVID-19 CXR severity scoring systems based on these findings.

**Methods:**

Admission frontal CXRs of 100 consecutive adults with proven COVID-19 pneumonia were analysed for technical features (projection, inspiratory excursion, exposure, vertical rotation) and pulmonary opacification. Descriptive statistics were evaluated. Kendall’s coefficient of concordance was used to assess observer agreement. One-way analysis of variance (ANOVA) was used to determine the association between technical factors and opacification. Levene’s test for homogeneity-of-variance and the Welch test were conducted where applicable. For inspiratory excursion, correlations were also calculated. Findings informed a review of published COVID-19 CXR severity scoring systems.

**Results:**

Poor inspiratory excursion obscured the lower lung zones in 41 cases and was associated with increased opacification in the visible (mid and upper) zones. Under- (*n* = 13) and over-penetration (*n* = 39) were associated with increased and decreased basal opacification, respectively. Eighty cases (80%) showed vertical rotation, without significant impact on opacification. Re-appraisal of published COVID-19 CXR scoring systems revealed that all simply assessed the visible lung fields, without considering inspiration or penetration.

**Conclusion:**

Published COVID-19 CXR severity scoring systems could be refined by appropriate consideration of inspiratory excursion and penetration. More broadly, technical considerations should inform all CXR interpretation.

**Contribution:**

This study advocates incorporating technical factor assessment into CXR severity scoring systems.

## Introduction

Coronavirus disease 2019 (COVID-19) was declared a global pandemic in February 2020. Within the first months of the pandemic, no fewer than 34 manuscripts described the computed tomography (CT) features of COVID-19 pneumonia.^1^ These early reports suggested that CT was the imaging examination of choice, yielding greater sensitivity and specificity than the chest X-ray (CXR).^1^ However, CT is not widely available in resource-limited settings.

While the CXR has lower sensitivity for the earliest COVID-19 pulmonary abnormalities, it can be used for monitoring progression, especially in the critically ill.^2^ Leading radiological societies therefore recommended chest radiography as the initial imaging investigation for COVID-19 pneumonia, in combination with reverse-transcription polymerase chain reaction (RT-PCR) tests.^2,3^ Chest X-rays, particularly mobile studies, thus played a pivotal role in the assessment of COVID-19 pneumonia, allowing repeated examinations at the point of care, while minimising transport-related cross-infection.^4^ A small but important series of early manuscripts defined the typical CXR features as bilateral, symmetrical pulmonary opacification with peripheral and basal predominance.^4,5,6,7,8,9,10,11^

Coronavirus disease 2019 CXR severity scoring systems have been shown to correlate with patient outcomes, including intensive care unit admission and death, prompting their use in patient triage and decisions regarding escalation of care. Seven such systems were described in five countries, across three continents (North America, Europe and Australia), and are summarised in Table 1.^12,13,14,15,16,17,18^ Two systems were developed prior to the COVID-19 pandemic^12,13^ and subsequently adapted, while the remainder^14,15,16,17,18^ evolved in response to the pandemic. With a single exception,^13^ all systems were developed with radiologist input; five had a radiologist as both first and senior author,^14,15,16,17,18^ and four were authored exclusively by radiologists.^14,15,17,18^ Four systems were published in radiological journals,^14,15,16,17^ two in journals of general medical interest^12,18^ and one in a respiratory journal.^13^ Although systems differ in the specifics of pulmonary scoring, they all assign a numerical value to the extent of pulmonary opacification, with higher scores denoting increased severity and poorer outcome.

**TABLE 1:** Chest X-ray severity scoring systems.

Maximum score	Name of system	Manuscript title	First author	Country of origin	Journal	Date of acceptance for publication	Landmarks defining pulmonary zones	Scoring system for lung involvement	Radiological terminology defined (Y/N)	Impact of technical factors assessed (Y/N)
5 points	5-point CXR scoring system for severe acute respiratory infection (SARI)	A chest radiograph scoring system in patients with severe acute respiratory infection: a validation study	Emma Taylor	New Zealand	*British Medical Journal*	16 December 2015	Nil	1 = normal; 2 = patchy atelectasis +/or hyperinflation +/or bronchial wall thickening; 3 = focal consolidation; 4 = multifocal consolidation; 5 = diffuse alveolar changes.	N	N
48 points	Scoring system for the radiological assessment of lung oedema (RALE)	Severity Scoring of Lung Oedema on the Chest Radiograph Is Associated with Clinical Outcomes in ARDS	Melissa Warren	US	*Thorax*	14 May 2018	The vertebral column and horizontally by the first branch of the left main bronchus.	Each quadrant scored on percentage opacification: 0 = nil; 1 = < 25%; 2 = 25% – 50%; 3 = 50 – 75%; 4 = > 75%, then a score (0–3) for overall density of alveolar opacities. Final score: sum of the product of the opacification and density scores for each quadrant (0–48).	N	N
18 points	Brixia Chest X-ray Severity Scoring System	COVID-19 outbreak in Italy: experimental chest X-ray scoring system for quantifying and monitoring disease progression	Andrea Borghesi	Italy	*Radiological Medicine*	13 April 2020	Upper zone: above inferior wall aortic arch; Mid-zones: from inferior wall aortic arch to inferior wall right inferior pulmonary vein. Lower zones: below inferior wall right inferior pulmonary vein. If anatomical landmarks not identified, each lung divided into three equal zones.	0 = no abnormalities, 1 = interstitial infiltrates, 2 = interstitial and alveolar infiltrates with interstitial predominance, 3 = interstitial and alveolar infiltrates with alveolar predominance. Total score: sum of all the six lung zones (0–18).	N	N
6 point	Chest Radiograph Severity Score	Clinical and Chest Radiography Features Determine Patient Outcomes in Young and Middle-aged Adults with COVID-19	Danielle Toussie	US	*European Radiology*	11 May 2020	Lower zone: costophrenic sulcus to inferior hilar markings. Middle zone: inferior hilar markings to superior hilar markings. Upper zone: superior hilar markings to the apices.	Each zone scored according to opacification 0 = absent; 1 = present and scores summed for total (0–6).	N	N
Percentage	Percentage of lung involvement	Chest X-ray for predicting mortality and the need for ventilatory support in COVID-19 patients presenting to the emergency department	Maurizio Balbi	Italy	*European Radiology*	13 September 2020	Distribution of ground glass opacities and consolidation classified as: (1) mainly peripheral one-third of lung or mainly central two-thirds of lung, or neither; (2) unilateral or bilateral; (3) upper zone (above inferior wall aortic arch) or middle zone (between inferior wall aortic arch and right inferior pulmonary vein) or lower zone (below right inferior pulmonary vein), or no predominance.	CXRs showing abnormalities other than GGOs and consolidation scored as zero. Pure consolidation scored as 3. The number of involved zones recorded. The overall extent of GGOs and consolidation assessed by visually estimating and then averaging the percentage of involvement within each lung.	N	N
24 point	A 24-point system for admission CXRs	Performance of a Severity Score on Admission Chest Radiography in Predicting Clinical Outcomes in Hospitalised Patients With Coronavirus Disease (COVID-19)	Russell A. Reeves	US	*American Journal of Radiology*	17 October 2020	Each lung divided into an upper and lower zone by equally dividing the hila into upper and lower segments in the craniocaudal dimension.	The extent of disease in each of the four zones scored: 0 = no disease, 1 = less than one-third, 2 = one-third to two-thirds, and 3 = more than two-thirds. Severity of disease in each zone scored (0 = normal, 1 = airspace opacification that does not obscure bronchovascular markings, 2 = airspace opacification that partially obscures bronchovascular markings, and 3 = complete opacification of the alveolar and interstitial spaces). The extent and severity scores summed over the four lung regions (right upper, right lower, left upper, and left lower) to create a composite score for each CXR (0–24).	N	N
12 point	Pennine score	Chest x-ray scoring as a predictor of COVID-19 disease; correlation with comorbidities and in-hospital mortality	Aparajita Singh	Scotland	*Scottish Medical Journal*	26 June 2021	CXR divided into three equal zones on each side, six parts in total.	A score of 0–2 assigned to each of the six parts of the CXR according to severity of lung findings. 0 – no lung opacity. 1 – ground glass opacity. 2 – consolidation.	N	N

COVID-19, coronavirus disease 2019; CXR, chest X-ray; GGOs, ground glass opacity; US, Unite States.

Despite these systems having been shown to be predictive of disease severity and poor outcome, none consider the impact of technical factors when assigning scores. For example, none defines the pulmonary zones by bony landmarks. They thus do not control for the extent of inspiratory excursion. Similarly, in no instance does the original description of the scoring system include details on the technical characteristics of the image cohort used to define the system. No details are provided regarding the adequacy of inspiratory excursion, radiographic penetration or patient positioning. This is surprising, because the criteria for CXR technical adequacy are well defined. Furthermore, technical factors are known to impact radiographic findings and have been implicated in spurious CXR interpretation.^19^ For example, poor inspiratory excursion increases the extent of pulmonary opacification, particularly in the bases.^19^ Additionally, mobile, supine CXRs in critically ill patients tend to have poor inspiratory excursion.^20^ Similarly, inadequate radiographic penetration accentuates pulmonary opacification, while the converse is true for over-penetration.^20^ Rotation on the sagittal plane increases hemithorax radiolucency on the side towards which the patient is turned, thereby decreasing opacification, while accentuating hemithorax opacification on the side from which the patient is turned.^21^

The premise of this study was that image quality should be an inherent standard of investigation and, where compromised, should be explicitly considered during interpretation.

The aim was thus to describe the technical features of COVID-19 pneumonia CXRs, determine their potential impact on CXR interpretation, and to reappraise published COVID-19 CXR severity scoring systems in the light of these findings.

## Research methods and design

### Data collection

A retrospective study was conducted at Tygerberg Hospital (TBH), Cape Town, South Africa (SA). A customised search of the institutional picture archiving and communication system-radiology information system (PACS-RIS) and electronic medical record (EMR) was conducted to identify the first 100 (*n* = 100) consecutive adults (18 years or older) undergoing CXR on admission to TBH for proven COVID-19 pneumonia, based on oro- or nasopharyngeal swab RT-PCR testing. Follow up CXRs for COVID-19 pneumonia were excluded.

The admission frontal CXRs of all subjects were independently analysed by two academic radiologists, with 20 and 35 years’ experience, respectively, utilising a customised forced-choice reporting template. Readers did not manipulate the brightness or contrast of the retrieved images and were blinded to clinical details other than the confirmed diagnosis of COVID-19. Each lung was divided into six zones by initial division into thirds: the *upper third* above an imaginary horizontal line joining the antero-inferior ends of the 2nd ribs; the *lower third* below such a line joining the antero-inferior ends of the 4th ribs, with the *middle third* between the lines.

**FIGURE 1:**
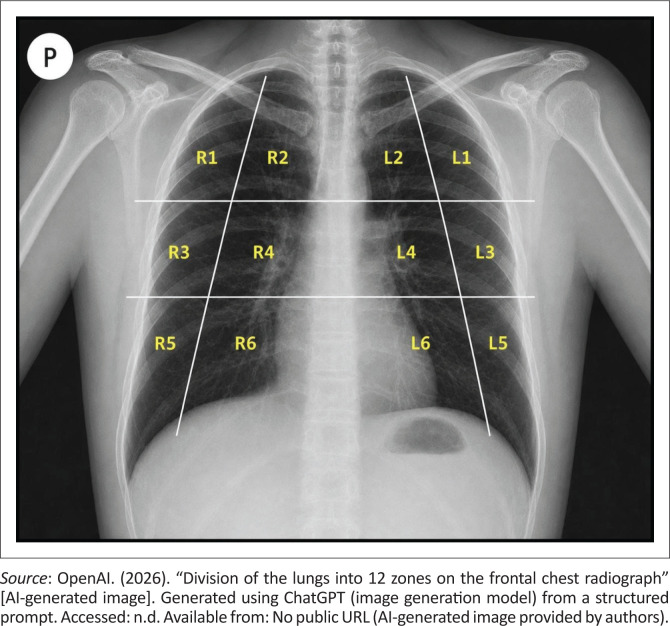
Division of the lungs into 12 zones on the frontal chest radiograph.

Each third was then bisected by an oblique line extending from the midpoint of the 1st rib, posteriorly, to the midpoint of the hemidiaphragm (Figure 1). The use of bony landmarks to define the initial six lung zones is unique to this study. All other systems define zones by soft tissue landmarks and are thus limited by being dependent on inspiratory excursion. The subdivision of the upper, middle and lower lung zones into medial and lateral components, yielding 12 zones in total, was aligned with the modified Brixia scoring system.^22^

The percentage opacification in each lung zone was scored in accordance with the radiological assessment of lung oedema (RALE) study,^13^ as follows: 0 (0%), 1 (1% – 24%), 2 (25% – 49%), 3 (50% – 74%) and 4 (75% – 100%) or ‘unsure’ where technical factors precluded optimal visualisation of a zone.

Key technical factors were evaluated as follows:

- **Inspiratory excursion:** Assessed by the number of anterior ribs above the right hemidiaphragm in the midclavicular line. Three anterior ribs were recorded as markedly decreased, four as suboptimal, five as satisfactory and six as optimal.^23^
- **Penetration:** Deemed optimal if the vertebral column was clearly demonstrated behind the heart, without differentiation of intervertebral disc spaces. Inability to discern the vertebral column behind the heart constituted under-penetration, while demonstration of the vertebral column with clear differentiation of the intervertebral disc spaces was recorded as over-penetration.^20^
- **Sagittal rotation:** The distance between the medial end of each clavicle and the adjacent vertebral spinous process was assessed. If the clavicle ends were equidistant, there was no rotation. Where there was a discrepancy, rotation was towards the side of the greater distance.^20^

### Statistical analysis

Detailed descriptive statistics were collated. Inter-observer agreement for the extent of pulmonary opacification in each lung zone was assessed using Kendall’s coefficient of concordance. The impact of inspiratory excursion, penetration and sagittal rotation on the extent of pulmonary opacification in each lung zone was compared using the one-way analysis of variance (ANOVA). Levene’s test was conducted to assess homogeneity-of-variance, and in cases where this assumption was not adhered to, the Welch test was conducted. For inspiration excursions, which were ordinal, correlations were also calculated.

### Ethical considerations

Ethical clearance to conduct this study was obtained from the Stellenbosch University Health Research Ethics Committee (No. N20/05/018_COVID-19). The study was approved by the National Research Database of the Department of Health. Anonymity of subjects was assured by assignment of a unique study number, known only to the Principal Investigator. All information was stored in a digital archive, with access password-protected and limited to co-authors.

## Results

### Patient demographics

The cohort had a median age of 49 years (interquartile range [IQR]: 39, 60) and included 65 female patients.

### Technical features

Study radiographs (*n* = 100) were taken from 25 March through 11 May 2020; eight (*n* = 8, 8%) were posterior-anterior (PA) erect projections, 62 (*n* = 62, 62%) sitting antero-posterior (AP) and 29 (*n* = 29, 29%) supine AP. There was a single PA prone view.

Four CXRs (*n* = 4, 4%) were satisfactory in all technical respects. Approximately one-third (*n* = 34, 34%) had one technical limitation, almost half (*n* = 48, 48%) had two, and 14 (*n* = 14, 14%) were deficient in all respects.

Overall, CXRs showed an average of 4.6 right anterior ribs (standard deviation [s.d.]: 0.83) above the right hemidiaphragm; nine (*n* = 9) showed six, half (*n* = 50) showed five, just over one quarter (*n* = 28) showed four and 13 (*n* = 13) showed three. Thus, poor inspiratory excursion obscured the lower lung zones (R5, R6, L5, L6) in 41 cases (41%).

Over- (*n* = 39) or under-penetration (*n* = 13) was present in a total of 52 cases (52%). Eighty cases (80%) showed sagittal rotation (right = 44; left = 36).

### Pulmonary opacification

**FIGURE 2:**
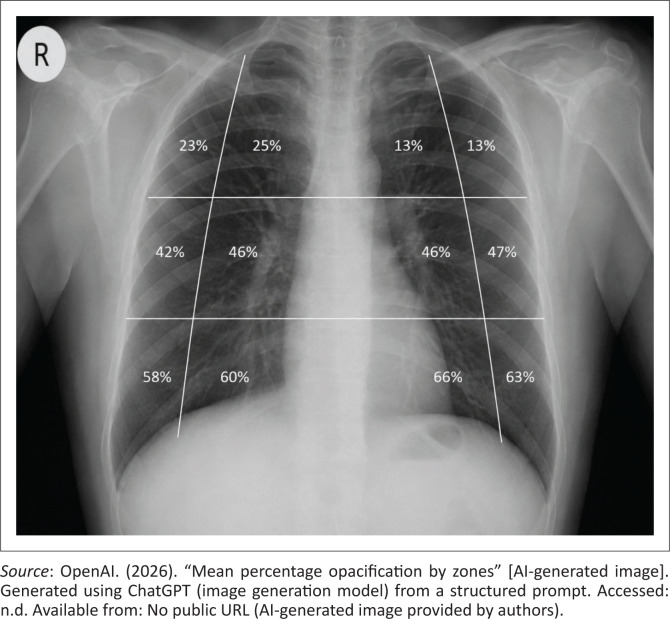
Mean percentage opacification by zones.

Fourteen CXRs (*n* = 14) showed no opacification, two (*n* = 2) opacification in a single zone, and 84 (*n* = 84) multifocal opacification. The mean percentage opacification was greatest in the basal zones (R5, R6, L5 and L6) with a bilateral, symmetrical distribution and an apical to basal gradient, but without peripheral accentuation (Figure 2).

### Scoring system analysis

The Kendall’s coefficient of concordance (0.71 to 0.97) showed strong to very strong observer agreement for the extent of pulmonary opacification across the 12 pulmonary zones.

The radiographic projection did not significantly impact inspiratory excursion. The average number of right anterior ribs visible above the hemidiaphragm was 4.75 (s.d.: 0.46) for PA erect, 4.63 (s.d.: 0.85) for AP sitting and 4.31 (s.d.: 0.85) for AP supine projections (*p* = 0.18).

Poor inspiratory excursion was associated with increased opacification in the mid- and upper zones (R1-R4, L1-L4) (Table 2). This is exemplified by R1, which showed a negative Pearson correlation coefficient (−0.27, *p* < 0.01) with an incremental increase in average opacification, from 0%, through 21%, 25% and 46% with six, five, four and three anterior ribs above the hemidiaphragm, respectively.

**TABLE 2:** Evaluation of inspiratory excursion.

Zone	% opacification										*F*-test	LSD†/Welch‡	Pearson	*p*-value
	Overall		3 anterior ribs		4 anterior ribs		5 anterior ribs		6 anterior ribs					
	Mean	s.d.	Mean	s.d.	Mean	s.d.	Mean	s.d.	Mean	s.d.				
R1	23.4	36.0	44.5^a1^	43.1	24.7^a2^	36.1	21.4^a3^	34.9	0.0^b^	-	0.03	a^1^,a^2^ = 0.17‡ a^1^,a^3^ = 0.09 a^2^,a^3^ = 0.70 a^1-3^,b < 0.01	−0.27	< 0.01
R2	24.5	31.3	42.7^a1^	31.8	20.8^b1^	30.1	26.3^a2b2^	32.0	0.0^c^	-	0.01	a^1^,b^1^ = 0.05‡ a^1^,a^2^ = 0.12 a^1-2^,c < 0.01 b^1-2^,c < 0.01	−0.23	0.02
R3	41.8	37.9	47.0^a1b1^	38.8	49.0^a2^	38.9	41.1^a3b2^	37.6	16.8^b3^	29.4	0.16	a^1^,a^2^ = 0.88† a^1^,a^3^ = 0.64 b^1^,b^3^ = 0.08 a^2^,b^3^ = 0.03	−0.18	0.07
R4	46.2	36.9	64.0^a1^	31.0	53.5^a2^	37.1	44.3^a3^	36.0	12.6^b^	28.8	< 0.01	a^1^,a^2^ = 0.40† a^1^,a^3^ = 0.10 a^3^,b = 0.10 a^1-2^,b < 0.01	−0.31	< 0.01
R5	58.4	38.3	-	-	60.3	38.2	47.5	39.1	-	-	0.36	-	−0.12	0.36
R6	60.1	36.5	-	-	61.9	36.1	50.3	2.0	-	-	0.39	-	−0.11	0.39
L1	12.5	23.5	30.1^a1^	32.2	10.8^a2^	24.1	11.1^a3^	20.6	0.0^b^	0.0	0.02	a^1^,a^2^ = 0.07‡ a^1^,a^3^ = 0.06 a^2^,a^3^ = 0.95 a^1-3^,b < 0.01	−0.27	< 0.01
L2	13.2	22.2	29.2^a1^	26.3	12.6^b1^	22.4	11.7^b2^	21.1	0.0^b3^	0.0	0.01	a^1^,b^1^ = 0.02† a^1^,b^2^ < 0.01 b^2^,b^3^ = 0.13 a^1^,b^1-3^ < 0.01	−0.28	< 0.01
L3	47.0	36.2	71.6^a1^	30.4	42.3^b1^	35.9	47.8^b2^	35.3	15.4^c^	30.0	< 0.01	a^1^,b^1^ = 0.01† a^1^,b^2^ = 0.03 b^1^,b^2^ = 0.5 a^1^,c^1^ < 0.01	−0.27	< 0.01
L4	46.0	35.1	77.3^a1^	25.0	48.6^b1^	35.3	41.8^b2^	32.0	12.6^c^	28.8	< 0.01	a^1^,b^1^ < 0.01† a^1^,b^2^ < 0.01 b^2^,b^3^ = 0.37 a^1^,c < 0.01	−0.41	< 0.01
L5	63.0	35.8	88.0	0.0	59.2	36.4	64.7	35.5	50.3	41.2	0.43	-	−0.14	0.26
L6	66.0	32.7	88.0	0.0	66.7	29.0	67.8	32.9	49.0	37.3	0.27	-	−0.19	0.11

Note: For each row, pulmonary zones with a different alphabetical superscript (a, b, c) had significantly different opacification. For example, in the case of L4, the mean percentage opacification of markedly under-inspired radiographs (3 anterior ribs, 77.3%, a^1^) was significantly more than that of poorly inspired radiographs (4 anterior ribs, 48.6%, b^1^, *p* < 0.01), satisfactorily inspired radiographs (5 anterior ribs, 41.8%, b^2^, *p* < 0.01) and optimally inspired radiographs (6 anterior ribs, 12.6%, c, *p* < 0.01). Additionally, there was a significant negative Pearson correlation coefficient with increasing inspiratory excursion (-0.41, *p* < 0.01).

s.d., standard deviation; LSD, Least Significant Difference test.

† , Least Significant Difference test;

‡ , Welch test.

Under- (*n* = 13) or over-penetration (*n* = 39) was associated with increased or decreased basal opacification (R5-6, L5-6), respectively. For example, average R5 opacification was 88% for under- and 49% for overpenetrated films, respectively (Welch test: *p* < 0.01) (Table 3).

**TABLE 3:** Evaluation of chest X-ray penetration.

Zone	% opacification								*F*-test	LSD†/ Welch‡
	Overall		Under-penetration		Optimal penetration		Over-penetration			
	Mean	s.d.	Mean	s.d.	Mean	s.d.	Mean	s.d.		
R1	23.4	36.0	27.2^a1^	33.1	28.8^a2^	40.0	15.5^a3^	30.5	0.20	a^1^,a^2^ = 0.90‡ a^1^,a^3^ = 0.28 a^2^,a^3^ = 0.08
R2	24.5	31.3	36.9^a1^	34.5	26.0^a2^	30.6	18.4^a3^	30.3	0.17	a^1^,a^2^ = 0.27† a^1^,a^3^ = 0.07 a^2^,a^3^ = 0.26
R3	41.8	37.9	52.5^a1^	35.8	45.8^a2^	38.2	33.6^a3^	37.4	0.19	a^1^,a^2^ = 0.59† a^1^,a^3^ = 0.13 a^2^,a^3^ = 0.14
R4	46.2	36.9	55.5^a1^	41.2	49.1^a2^	35.7	40.1^a3^	36.9	0.35	a^1^,a^2^ = 0.59† a^2^,a^3^ = 0.26 a^1^,a^3^ = 0.21
R5	58.4	38.3	88.0^a^	0.0	61.2^b1^	37.9	49.0^b2^	39.9	0.05	a,b^1, 2^ < 0.01‡ b^1^,b^2^ = 0 .27
R6	60.0	36.5	88.0^a^	0.0	60.7^b1^	35.0	52.9^b2^	39.0	0.07	a,b^1-2^ < 0.01‡ b^1^,b^2^ = 0.45
L1	13.0	23.5	17.4^a1^	33.0	10.8^a2^	19.3	13.0^a3^	24.9	0.66	a^1^,a^2^ = 0.37† a^2^,a^3^ = 0.67 a^1^,a^3^ = 0.56
L2	13.0	22.2	13.6^a1^	17.5	14.2^a2^	23.6	11.7^a3^	22.6	0.87	a^1^,a^2^ = 0.94† a^2^,a^3^ = 0.61 a^1^,a^3^ = 0.79
L3	47.0	36.2	61.0^a1^	35.6	46.1^a2^	37.5	42.0^a3^	34.3	0.27	a^1^,a^2^ = 0.19† a^2^,a^3^ = 0.6 a^1^,a^3^ = 0.1
L4	46.0	35.1	52.3^a1^	38.6	48.5^a2^	33.8	40.0^a3^	35.5	0.41	a^1^,a^2^ = 0.74† a^2^,a^3^ = 0.26 a^1^,a^3^ = 0.28
L5	63.0	35.8	88.0^a^	0.0	60.8^b1^	38.6	59.8^b2^	35.5	0.15	a,b^1-2^ < 0.01‡ b^1^,b^2^ = 0.18
L6	66.0	32.7	88.0^a^	0.0	63.7^b1^	32.9	63.6^b2^	34.8	0.18	a,b^1-2^ < 0.01‡ b^1^,b^2^ = 0.99

Note: For each row, zones with a different alphabetical superscript (a, b) had significantly different opacification. For example, in the case of zone R5, the mean percentage opacification on underpenetrated images (88%, a) was significantly higher than that for optimally penetrated images (61.2%, b^1^; *p* < 0.01) and overpenetrated images (49%, b^2^; *p* < 0.01). Conversely, the mean percentage opacification of optimally penetrated images (61.2%; b^1^) was not significantly different from that of overpenetrated images (49%, b^2^; *p* = 0.27).

s.d., standard deviation; LSD, Least Significant Difference test.

† , Least Significant Difference test;

‡ , Welch test.

Sagittal rotation did not significantly impact opacification (Table 4).

**TABLE 4:** Evaluation of vertical rotation.

Zone	% opacification								*F*-test
	Overall		Rotation to right		No rotation		Rotation to left		
	Mean	s.d.	Mean	s.d.	Mean	s.d.	Mean	s.d.	
R1	23.4	36.0	26.1	38.5	20.2	33.2	22.1	34.9	0.80
R2	25.0	31.3	23.0	29.6	22.7	30.7	27.3	34.1	0.80
R3	42.0	37.9	40.8	38.6	44.1	36.5	41.6	38.8	0.95
R4	46.2	36.9	43.5	36.8	40.9	37.5	52.5	36.7	0.44
R5	58.4	38.3	52.7	41.7	59.2	39.8	62.9	35.1	0.68
R6	60.0	36.5	50.9	39.6	59.2	39.8	68.6	30.7	0.27
L1	13.0	23.5	12.6	24.9	9.5	23.4	14.1	22.2	0.78
L2	13.0	22.2	11.8	21.1	10.1	21.8	16.5	23.8	0.51
L3	47.0	36.2	45.8	36.0	38.5	36.0	51.7	36.7	0.42
L4	46.0	35.1	42.6	34.6	39.7	36.3	52.8	34.8	0.30
L5	63.0	35.8	55.9	39.3	65.1	36.4	69.1	31.4	0.39
L6	66.0	32.7	59.1	36.1	60.0	35.0	72.1	26.8	0.37

s.d., standard deviation.

## Discussion

To our knowledge, this study represents the only assessment of the impact of technical factors on COVID-19 scoring systems. Our finding that image quality is compromised by technical factors which are not explicitly considered in current scoring systems makes an important contribution to the body of knowledge in this domain. Furthermore, the findings are broadly applicable, with relevance to all CXR scoring systems. Our findings serve as a reminder that basic radiographic principles should be rigorously applied, regardless of clinical pressures and academic imperatives.

Quality assurance is the cornerstone of diagnostic radiology. Objective criteria have been defined for optimal CXR quality and are applicable to all clinical settings. Early CXR literature discussed exposure optimisation for visualisation of the pulmonary parenchyma.^24^ The influence of inspiratory excursion on the radiographic appearance of the lung was first documented in the *Lancet* of 27 June 1903 by Dr J.F. Halls Dally, Assistant Resident Medical Officer at the Royal National Hospital for Consumption. He reported that ‘the lungs brighten on deep inspiration because of the greater amount of contained air’ and suggested the term ‘transradiancy’ be used to describe this appearance.^25^ However, it was another three-quarters of a century before the landmark 1978 manuscript in *Clinical Radiology* elucidated the physics behind the transradiancy of the hemithorax resulting from sagittal rotation.^21^

This manuscript provides insights into the technical challenges of COVID-19 chest imaging, typically involving emergency, mobile studies on compromised patients, requiring strict infection control. More than 90% of CXRs were AP projections (rather than the conventional erect PA views), a large majority (80%) showed vertical rotation, more than half (52%) suboptimal exposure and a substantial proportion (41%) inadequate inspiration, while only 4% achieved optimal quality. By comparison, a 2022 study in a large Nepalese teaching hospital, which assessed general CXR quality in an undifferentiated population, including ambulant patients and routine investigations, showed optimal quality in 22%, rotation in 44%, poor exposure in 18% and inadequate inspiration in just 7%.^26^

Because all COVID-19 CXR scoring systems simply assess the visible lung fields, without control for inspiratory excursion or radiographic exposure, all have inherent bias towards increased opacification on poorly inspired or under-penetrated projections. Conversely, they have a bias towards decreased opacification on over-exposed films.

Further work is required to better understand the impact of technical quality on CXR severity scoring systems. Manuscripts describing such systems should ideally include an analysis of the sample’s technical characteristics, as well as the post-processing functionality used when viewing images. Such detail is important, because modern PACS allow manipulation of brightness and contrast, which can mitigate the effects of over- and under-exposure. However, no image manipulation can compensate for inadequate inspiratory excursion. Going forward, scoring systems would be enhanced by rigorously assessing inspiratory excursion based on bony thoracic landmarks (ribs), in accordance with the principles of conventional chest radiographic quality assurance. Furthermore, scoring systems should ideally incorporate a weighting factor for technical factors.

Strengths of this study included the broad alignment of the percentage pulmonary opacification reporting methodology with published CXR severity scoring systems. Additionally, the extensive experience of the reporting radiologists contributed to strong inter-observer agreement.

Furthermore, the rigour and granularity of the zonal statistical analysis offer a unique contribution to the discourse on CXR interpretation. To the best of our knowledge, this is the first quantitative assessment of the impact of technical factors on zonal pulmonary opacification. As such, our findings provide the first description of the differential zonal impact of CXR technical parameters. It is hoped that this study will provide a framework for further similar analyses.

The study was limited by the retrospective design and small sample. Notwithstanding the latter, statistical significance was demonstrated for key technical parameters in specific pulmonary zones. A larger cohort may have shown that changes in exposure significantly impact mid- and upper-zone opacification. Although reporting methodology did not include the nature of zonal opacification (interstitial *vs*. alveolar infiltrates), which was included in four severity scoring systems,^13,14,16,17^ it is unlikely that such additional data would have impacted the key findings. The study assumed subjects had normal pulmonary compliance, being the measure of lung expansion per unit increase in transpulmonary pressure. This was considered appropriate, because the study was confined to admission radiographs. Quality assurance principles are founded on the patient’s capacity to achieve optimal inspiratory excursion. Of note, four of the seven scoring systems also involved admission radiographs.^13,14,17,18^

It is hoped that this study will reinforce the role of technical factors in the accurate interpretation of CXRs and stimulate the development of CXR severity scoring systems for acute chest pathology that include technical factor assessment.

## Conclusion

Published COVID-19 CXR severity scoring systems could be refined by consideration of inspiratory excursion and penetration. More broadly, technical considerations should inform all CXR interpretation.
